# Influence of Pulsed Interference Laser Heating on Crystallisation of Amorphous Fe_77_Cu_1_Si_13_B_9_ Ribbons

**DOI:** 10.3390/ma17092060

**Published:** 2024-04-27

**Authors:** Agnieszka Radziszewska, Olaf Czyż

**Affiliations:** 1 Faculty of Metals Engineering and Industrial Computer Science, AGH University of Krakow, al. Mickiewicza 30, 30-059 Krakow, Poland; 2 MAKE IT Olaf Czyz, Zieleniec 61A, 46-034 Pokoj, Poland; o.czyz@o2.pl

**Keywords:** amorphous alloy, pulsed laser interference heating, FeCuSiB ribbons, magnetic field lines, two-dimensional crystallised micro-area

## Abstract

Amorphous Fe_77_Cu_1_Si_13_B_9_ ribbons were treated with pulsed laser interference heating (PLIH). The research results will significantly contribute to a better understanding of the impact of PLIH on crystallisation and magnetic properties in precisely defined micro-areas of Fe_77_Cu_1_Si_13_B_9_ (FeCuSiB) ribbons, which has not yet been described in the literature. It was confirmed here that the use of the laser heating process allowed for the achievement of two-dimensional crystallised micro-areas, periodically distributed (at a distance of 17 µm) on the surface of the amorphous ribbons. The correlation between structural changes (SEM, TEM, HRTEM) and the distribution of magnetic field lines of heated amorphous Fe_77_Cu_1_Si_13_B_9_ ribbons is presented. Particular attention is paid to structural changes in micro-areas where, by controlling the laser interference heating process, the partial crystallisation of amorphous alloys and the formation of clusters or single nanocrystallites (α-Fe(Si)) embedded in an amorphous matrix occur. The addition of copper to the FeSiB alloy promoted the inhibition of grain growth. Electron holography of micro-areas confirmed shifts in the magnetic field lines in the areas of nanocrystallites, the presence of which in the structure caused the magnetisation of the surrounding amorphous matrix.

## 1. Introduction

The very rapid continuous development of modern industries is focused on the desire to discover and understand, in particular the optimisation of nanocrystalline soft magnetic materials, in light of their subsequent applications and taking into account the significant current subject of reducing energy consumption and losses [1,2,3]. Research is ongoing on new materials with unique soft magnetic properties [4,5,6,7,8]. Since the middle of the twentieth century, there has been a huge increase in interest in nanocrystalline soft magnetic materials, which primarily play a significant role in the production and distribution of energy in the electronics industry, telecommunications, data storage, and current and magnetic field sensors [9,10,11,12]. Among soft magnetic materials, alloys of unalloyed iron and FeSiB with the addition of Cu and Nd are present in the largest amounts [13,14,15]. They have influenced significant progress in magnetism, where control of the microstructure and composition of the alloy plays a significant role. The benefits of the properties of FeSiB alloys can be achieved as a result of high solidification rates and an optimised annealing process. What distinguishes these types of alloys is their properties. They are characterised by high saturation induction, low magnetic coercivity, high magnetic permeability, and low loss. As a result of their favourable properties, the use of soft magnetic materials is expanding constantly. Produced by Yoshizawa et al. [16], a new alloy (with the composition Fe_73.5_Nb_3_Cu_1_Si_13.5_B_9_) has attracted the attention of researchers and industry due to its excellent magnetic soft properties, which are obtained as a result of a properly selected structure. FeCuSiB alloys (in the form of ribbons) are obtained by rapid solidification using the melt-spinning technique, which leads to the achievement of an amorphous structure and the desired properties. At the same time, note that the conventional annealing process of the amorphous phase, leading to the achievement of nanocrystalline material, is long-lasting and energy-intensive. The furnace annealing of the FeSiB alloy can lead to the formation of the α-Fe(Si) phase with a grain size of approximately 10 nm [17,18] and the precipitation of Fe_2_B, which independently crystallises from the amorphous matrix [3,19,20]. The addition of copper (1%) to the FeSiB alloy, as presented by Yoshizawa [21], to the amorphous phase contributes to the reduction in grain size, resulting in improved soft magnetic properties of the alloy, reduces the crystallisation temperature of the α-Fe phase, and provides places for primary crystallisation [2,22,23]. Inoue et al. [24,25] confirmed that amorphous alloys containing nanocrystallites in the structure show significantly higher strength compared to fully amorphous alloys.

Therefore, the attention paid to FeCuSiB alloys is reflected in the steady increase in the number of publications in the past decades and the great industrial interest in nanocrystalline soft magnetic materials. During alloy crystallisation, as the copper content in the alloy increases, the possibility of the number of nucleation sites increases, which was confirmed by Kulik [23]. At the same time, the conventional annealing of amorphous ribbons leads to complete crystallisation, loss of plastic properties, and high brittleness, consequently causing the destruction of the material. This significantly reduces the application of these materials. Indeed, it is necessary to treat the material in such a way that the heating of the ribbons leads to a partially nanocrystalline structure.

However, despite significant progress, to optimise devitrification, microstructure control remains a significant scientific and technological challenge.

To obtain a nanometric structure, very rapid cooling is necessary, at rates of up to 10^5^–10^6^ K·s^−1^. One of the most modern surface modification techniques is pulsed laser interference heating (PLIH). So far, the literature has not yet placed much emphasis on the characterisation of the microstructure of the FeCuSiB alloy obtained as a result of interference laser heating. This technique enables a change in the structure in micro-areas (dots) with a diameter of 10 μm, where nanocrystals occur.

The overlap of the incident beams on the material’s surface causes their interaction and the formation of one-dimensional (lines) and two-dimensional (dots) interference images. For example, the interference of two beams at an angle of 180° leads to one-dimensional images: lines. Adjusting the angle of incidence of the beams directly affects the period (distance) of occurrence of the areas. However, reducing the wavelength of laser radiation decreases the distance between the dots. The intensity of the interference field of the two beams is described by the following relationship (1) [26]:

(1) $$I\left(x\right)=I_{1}+I_{2}+2\sqrt{I_{1}I_{2}}\cos(2kx\;\sin\theta ),$$

where the following variables are used:

k—the wave vector of the laser beam;
x—position in the sample;
I_1_, I_2_—intensity of the laser beam;
θ—angle of incidence of the laser beam.

The formed micro-areas are regularly distributed on the surface of the material, while the distance between them depends on the parameters of the quartz prism applied for interference of the laser beams. The distance is described by the following Equation (2) [27]:

(2) $$\Lambda =\frac{\lambda }{2\;\sin(\sin^{-1}\left(\eta \sin\alpha \right)-\alpha )}$$

where the following variables are used:

α—refractive angle;
η—refractive index of quartz prism (1.459).

A review of the literature allows us to conclude that our work is the first to present the impact of the laser beam on FeCuSiB ribbons and their magnetic properties analysed using electron holography. Most of the authors deal with the laser heating of amorphous FeSiB and FeNbCuSiB alloys [28,29]. Recently, McKinstry et al. [28] presented the effect of a single linear laser path on changes in the structural and magnetic properties of an amorphous FeSiB film. The laser power used was 100 W, and the laser fluence was 0.42–0.91 J/mm. Scientists found that obtaining a laser fluence higher than 0.48 J/mm leads to partial crystallisation of the amorphous FeSiB layers, and a continuous increase in the α-FeSi fraction at laser fluence leads to an improvement in the saturation magnetostriction of the FeSiB layers [28]. However, the formation of Fe_2_B in significant amounts (2%) led to the loss of magnetic properties of the soft films. Nykyruy et al. [29] used continuous irradiation with a 1.06 µm laser with a laser power of 45 W to heat the structure of an amorphous Fe_73.5_Nb_3_Cu_1_Si_15.5_B_7_ ribbon. In their studies, the diameter of the laser spot was approximately 10 mm, and the exposure time was 0.25–0.70 s. The authors [29] determined that crystallisation began at 0.35 s with the formation of the α-Fe(Si) nanocrystalline phase, while after 0.55 s, two nanocrystalline phases were formed: α-Fe(Si) and the hexagonal H phase.

The distribution of structural elements as a result of the impact of the laser beam on the material depends on the process parameters, for example, the energy of the laser beam, the exposure time, and the frequency of the pulses. The use of parameter variables leads to changes in the temperature distribution in the material. This work is based on the literature reports that included temperature distribution models presented by Ashby and Esterling [30]. These issues were also developed by Bell [31] and other researchers [32].

The scientific community is expanding research on FeCuSiB materials because before new materials are developed, it is necessary first to understand the structural changes and magnetic field lines of amorphous Fe_77_Cu_1_Si_13_B_9_ ribbons subjected to local laser surface treatment. Therefore, an important goal of this research is to determine the possibility of creating a nanocrystalline structure in amorphous Fe_77_Cu_1_Si_13_B_9_ ribbons by the interaction of a laser beam, which allows for the achievement of precisely defined periodically arranged points (62,500) on their surface (5 × 5 mm), and to determine how the structure affects the distribution of magnetic field lines of force.

This work makes a significant contribution to the field of surface treatment of soft magnetic ribbons. The novelty of this work is the possibility of using a modern technique that leads to the attainment of nanocrystalline structures in precisely defined areas. The PLIH method enables the uniform millisecond heating of a 5 × 5 mm surface, which results in the ‘covering’/heating of large areas of the web. Otherwise, a nonuniform dot would result. This technique has many advantages over conventional annealing, including that the laser beam used heats part of the volume of the ribbon and does not cause its brittleness, as in the case of conventional annealing, shortens the time needed to create a nanocrystalline structure in the subsurface layer of the ribbons, and does not require the use of coolants or vacuum, as, e.g., in the case of electron techniques. Consequently, we significantly improve the efficiency of the process and reduce its costs.

As part of this work, detailed analyses of structural changes in Fe_77_Cu_1_Si_13_B_9_ ribbons after pulsed laser interference heating (PLIH) were performed using scanning electron microscopy (SEM) and transmission electron microscopy (TEM) techniques, selected area diffraction (SAD), and high-resolution transmission electron microscopy (HRTEM). Electron holography was used to determine local changes in the magnetic field in laser-heated micro-areas of the ribbons.

## 2. Materials and Methods

The materials provided for this research were 35 µm thick and 10 mm wide amorphous ribbons made of the Fe_77_Cu_1_Si_13_B_9_ alloy obtained by the melt-spinning method and supplied by Allied Signal Corp., Morristown, NJ, USA. Pulsed laser interference heating was performed using a Q-switch Nd:YAG laser at a laser radiation wavelength of 1064 nm. Laser heating was performed using the basic TEM_00_ laser mode and the Gaussian energy distribution of the laser beam. The amplification system allowed for a smooth adjustment of the pulse energy from 60 to 470 mJ, with a pulse duration of 10 ns. The parameters of the PLIH technique used were selected on the basis of structural changes caused by the modification of the energy or the number of pulses. The application of low energy led to the achievement of very thin layers up to 100 nm. An increase in the energy and the number of pulses led to too much melting and laser ablation processes (evaporation of the material). The changes obtained in the micro-areas corresponded to those obtained for FeSiB ribbons, which were discussed in our previous publications [6,33].

The interference system consisted of a quartz prism in the shape of a regular tetrahedral pyramid with an apex angle of 172°. Due to the use of a prism, interference of four laser beams was achieved. This allowed us to obtain a grid of 62,500 laser-heated micro-areas on a 5 × 5 mm surface. To determine surface morphology, the ribbons were electrolytically etched in a 10% solution of chromium III oxide (Cr_2_O_3_) with water, at a voltage of 10 V and a time of 10 s. Observations were made with a NovaNanoSEM 450 scanning electron microscope (SEM—FEI, Eindhoven, the Netherlands). SEM analysis was performed in the secondary electron (SE) modes.

**Figure 1:**
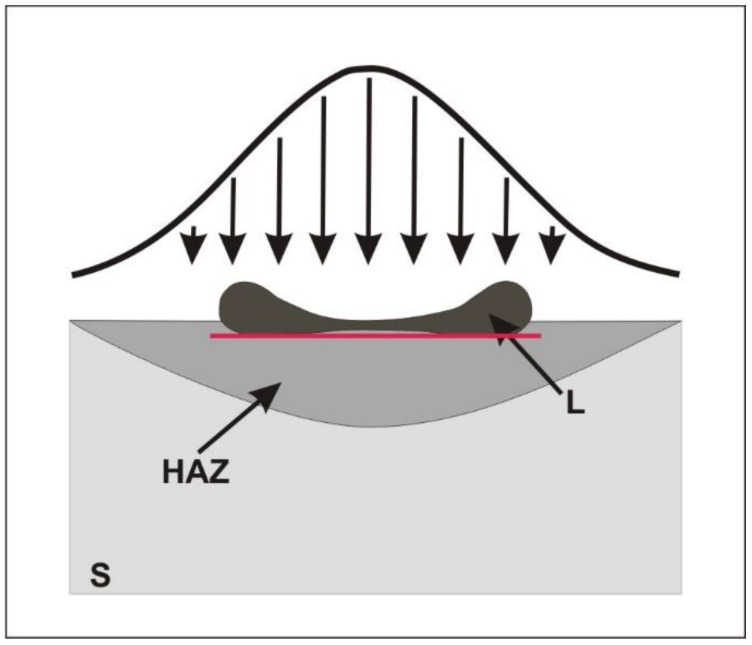
Position of the thin foil (red line) parallel to the laser-heated surface. Abbreviations of symbols: L—liquid; S—solid; HAZ—heat-affected zone; arrows–laser beam interaction.

Conventional TEM analysis was performed to determine microstructural and phase changes of the ribbons after laser processing using a JEOL JEM 200CX, Akishima, Japan. Due to the small volume of crystallised ribbon material, initial X-ray diffraction analysis showed the presence of an amorphous phase, so the focus was on the analysis of micro-areas using selected area diffraction (SAD-TEM) and high-resolution transmission electron microscopy (HRTEM). High-resolution electron microscopy (HRTEM) investigations of nanocrystalline areas embedded in an amorphous structure were carried out using a G2 F20 from Tecnai, Hillsboro, OR, USA. High-resolution images were analysed using GATAN Microscopy Suite software (GMS 3). Thin films for TEM examinations were prepared by cutting 3 mm disks from laser-heated areas. Then, thin films were made in two planes: parallel and perpendicular to the heated surface. In order to prepare the thin films in a plane parallel to the heated surface, the disks were ground from the heated side to remove the roughness created during laser heating. In the next step, the disks were mechanically polished in the central part on the laser-untreated side, using a GATAN 656 dimpler (Pleasanton, CA, USA). After dimpling, the disks were electro-polished using a STUERS TenuPol-5 polishing machine (Ballerup, Denmark). The polishing reagent was a 5% solution of perchloric acid (HClO_4_) in ethanol, the voltage was U = 38–41 V, and the temperature was below—24 °C. The disks were then cleaned using a Leica EM RES101 ion milling machine (Wetzlar, Germany) or PIPS from GATAN applying the following parameters: gun voltage U = 3 keV and gun angle—3°. This thin film preparation allowed us to analyse the changes in structure in the area marked with a red line in Figure 1.

**Figure 2:**
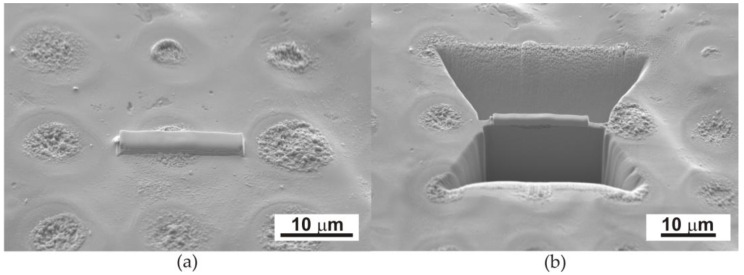
Scanning electron microscopy images showing the periodic dots distributed on the surface of the Fe_77_Cu_1_Si_13_B_9_ ribbon and the steps of lamella preparation (**a**,**b**) using the FIB method.

To prepare thin films in a plane perpendicular to the heated surface, lamellas were cut from the dot area using a focused ion beam (FIB) by SEM-FIB Quanta 3D from FEI (Eindhoven, the Netherlands) (Figure 2).

Electron holography made it possible to determine the distribution of magnetic field lines in laser-heated micro-areas. Therefore, this study was carried out using an HRTEM FEI Titan CUBED 80–300 microscope (Eindhoven, the Netherlands) on thin foils, using the Lorentz mode. Additionally, electron holograms were also analysed when a magnetic field was excited with an objective lens by flowing ±10% of the maximum lens current. Attempts were also made to test the hardness of the FeCuSiB ribbons. Due to the thickness of the ribbons, nanohardness tests were carried out at low loads (up to 5 mN). However, local deformations of the ribbons made it impossible to obtain reliable results.

## 3. Results

### 3.1. Microstructural Characterisation

#### 3.1.1. Scanning Electron Microscopy Examinations

**Figure 3:**
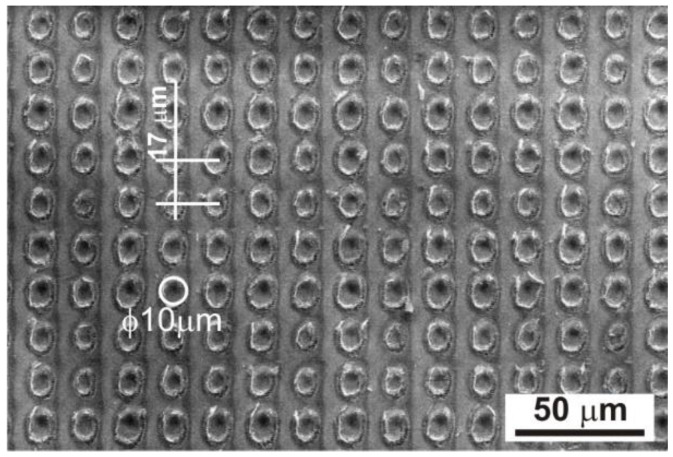
The SEM image shows regularly distributed dots formed on the surface of the Fe_77_Cu_1_Si_13_B_9_ ribbon laser-heated with an energy of 120 mJ and a number of pulses of 500.

The pulsed interference interaction of the laser beam with the amorphous ribbon led to the formation of periodically arranged micro-areas on a 5 × 5 mm surface, consisting of 62,500 dots. The periodic distribution of dots, obtained by laser heating with an energy of 120 mJ and a pulse number of 500, is shown in Figure 3 using the scanning electron microscope. Observations with a scanning electron microscope showed a two-dimensional structure of periodically distributed laser-heated micro-areas at a distance of approximately Λ = 17 µm. The diameter of the dots was approximately 10 μm (Figure 3).

**Figure 4:**
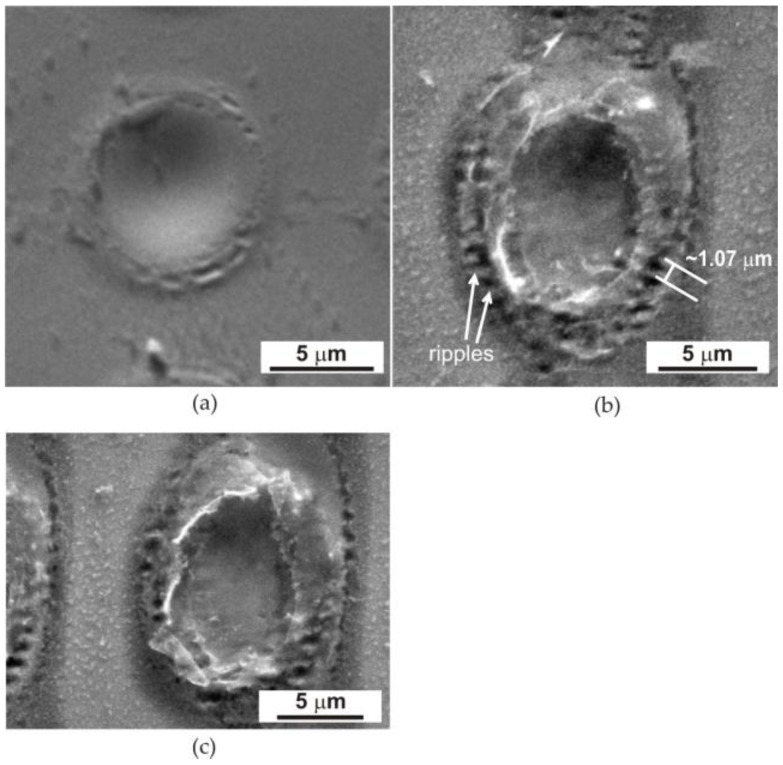
SEM images of the surface of the Fe_77_Cu_1_Si_13_B_9_ ribbon after pulsed laser interference heating with an energy of 120 mJ and a variable number of pulses, (**a**) 200 and (**b**) 500, and (**c**) an energy of 170 mJ and 10 pulses.

The increase in the number of laser pulses and energy led to the formation of a more macroscopically visible interference pattern on the surface of the amorphous ribbon, which is shown in Figure 4.

The structural changes created on the surface of the Fe_77_Cu_1_Si_13_B_9_ ribbons resulting from the use of variable energy and the number of pulses correspond to our previous results obtained for FeSiB [6,34]. Therefore, the use of a small number of pulses (10) and a lower beam energy (60–100 mJ) to heat the amorphous ribbon leads to the effect of heating its surface to a ribbon thickness of approximately 100 nm. However, increasing the energy to 120 mJ and using a larger number of pulses resulted in the melting of the ribbon surface, and at the same time, evaporation of the material in micro-areas occurred (Figure 4a,b). Heating with a higher energy of the laser beam (above 170 mJ) was responsible for the melting of the material, its partial ablation (evaporation), and the deformation of the edges of the heated micro-areas (Figure 4c). Note that the energy distribution in the laser beam is Gaussian, which affects the concentric distribution of individual zones of the structure (Figure 4). The highest energy occurs in the centre of the beam cross-section and decreases along its radius, causing a change in the structure in individual micro-areas. It is clearly visible that in the central part of the dot, with a smaller number of pulses of 200 and an energy of 120 mJ (Figure 4a), the phenomenon of the melting and ‘pushing’ of liquid material into the edge zones of the dot arises as a result of the dynamics of the process, the action of the shock wave, and the centrifugal force. As a result, an outflow of liquid material can be seen at the edges of the dots, as shown in Figure 1.

At the same time, it should be noted that, in the centre of the dot, the molten material has the highest temperature and is therefore the rarest, which facilitates its movement. If the number of pulses is higher (500) (Figure 4b), characteristic coarse ripples are formed [35], and they are the result of interference between the incident light of the laser beam and the surface scattering wave [35,36,37]. The period of the observed coarse ripples is close to the wavelength (1064 µm) of the incident Nd:YAG laser radiation (~1.07 µm) (Figure 4b).

Due to the structural effects presented above, the following sections of the TEM results will present a ribbon heated with a laser beam energy of 120 mJ and 500 pulses.

#### 3.1.2. TEM and HRTEM Examinations

**Figure 5:**
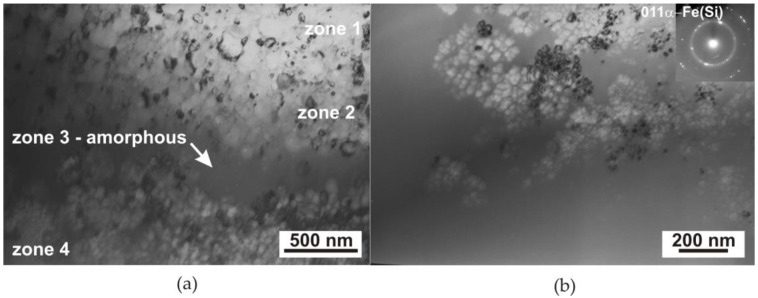
TEM plane-view image of an Fe_77_Cu_1_Si_13_B_9_ ribbon laser-heated with an energy of 120 mJ and 500 laser pulses, view of the central part of the micro-area/dot: (**a**) general view; (**b**) a peripheral zone—single and clusters of nanocrystals sounded by an amorphous matrix.

Observations using transmission electron microscopy showed the presence of a crystalline structure in the laser-heated micro-areas (Figure 5). Figure 5 shows TEM (BF) images in cross-section perpendicular to the ribbon surface of a single laser-heated micro-area of the Fe_77_Cu_1_Si_13_B_9_ ribbon. Due to the appearance of a temperature gradient in the areas heated by the laser beam, structural changes were observed in which four zones can be distinguished (Figure 5a). In the first zone, nanocrystalline grains are visible in the centre of the micro-area, around which zone 2 was formed. This zone was in contact with the liquid material; thus, the temperature reached was higher than in zone 1, resulting in grain growth. Subsequently, zone 3 with an amorphous Fe(Si, B) structure is present in the area of the solidified liquid, while in the peripheral area (zone 4), the grains were reduced to nanometric size (Figure 5b). In this zone, single nanocrystals (~10 nm) and their clusters (Figure 5b) surrounded by an amorphous matrix were observed. The existence of an amorphous structure of the ribbon was confirmed by annular electron diffraction; additionally, electron diffraction showed a small number of reflections from crystallised areas (inset in Figure 5b). The addition of copper to the FeSiB alloy, the initiation of crystallisation using laser heating, and immediate cooling facilitate the saturation of the structure with nanocrystallites α-Fe(Si). Furthermore, Cu segregates to the grain boundaries and blocks their growth, which was confirmed by Kulik’s results and those of other researchers [22,23,38].

Note that laser heating led to the initiation of the formation of crystalline areas, but too short a laser treatment time allowed only a slight increase in the recrystalline phase. This resulted in the formation of a structure composed of a large number of nanometric crystallites (Figure 5) that is not possible to obtain during traditional thermal treatment. The nanocrystalline structure enables an improvement in soft magnetic properties through low magnetic loss due to magnetisation reversal, high magnetic permeability, and low magnetostriction [39,40,41]. Changes in the magnetic properties of ribbons before and after laser heating (magnetic hysteresis loops, magnetic force microscopy) were discussed in our previous articles [6,33].

**Figure 6:**
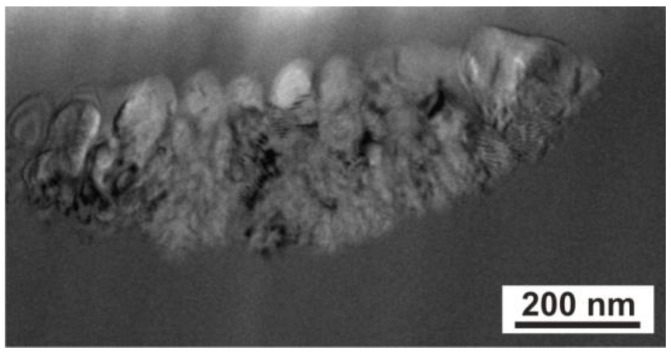
TEM image of an Fe_77_Cu_1_Si_13_B_9_ ribbon cross-section heated with a laser, showing a cluster of nanocrystallites in an amorphous matrix in the peripheral zone of the dot.

The laser heating of the Fe_77_Cu_1_Si_13_B_9_ alloy with an energy of 120 mJ and a large number of laser pulses (500) in the peripheral areas of the dots led to the formation of clusters of nanocrystallites in an amorphous matrix (Figure 6).

**Figure 7:**
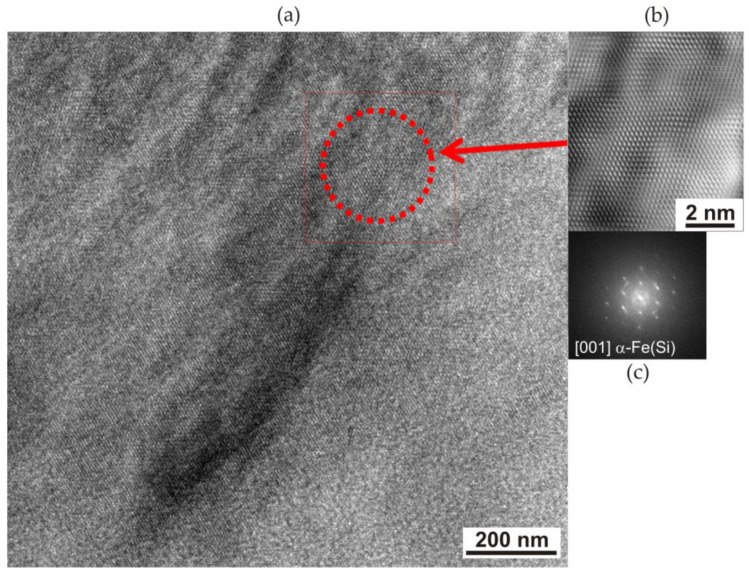
HRTEM image of an Fe_77_Cu_1_Si_13_B_9_ ribbon heated with an energy of 120 mJ and 500 laser pulses: (**a**) general view; (**b**) enlargement of the selected area from (**a**) (marked with the red arrow and square); (**c**) FTT pattern calculated from the area marked by the dotted circle on the (**a**).

The fast Fourier transform (FFT) of the area of the partially crystallised amorphous matrix indicates the presence of an α-Fe(Si) with a large amount of amorphous matrix (Figure 7). High-resolution transmission microscopy of the ribbons revealed the occurrence of an amorphous structure, confirmed by the presence of a halo ring shown in the fast Fourier transform (Figure 7c). The supply of energy, as a result of heating the material with a laser beam, contributed to the partial crystallisation of the amorphous matrix in the area of the boundary with the crystallites (Figure 7b). High-resolution TEM observations revealed a structure composed of nanocrystallite areas of the α-Fe(Si) phase with orientation [001] in an amorphous matrix (Figure 7c).

#### 3.1.3. Electron Holography: Distribution of Magnetic Force Lines

The use of electron holography made it possible to determine local changes in the magnetic field (contours of the solid magnetic phase) in an amorphous material, Fe_77_Cu_1_Si_13_B_9_ ribbons, crystallised as a result of pulsed interference laser heating, which was performed with a TEM FEI Titan CUBED 80-300 (Eindhoven, the Netherlands) using the Lorentz mode. The samples were prepared in thin foils for TEM. Holograms were obtained by applying the sample configuration ‘upside–downside’. Consequently, first, a hologram of the sample was performed in the upper configuration (upside); then, a hologram of the sample was created, which was rotated by 180° (downside configuration) relative to the holder axis in the microscope. The obtained holograms were computer-processed using the PyHoLo programme [42], which is necessary to separate the phase shift caused by electrostatic and magnetic field forces and was used to determine the magnetic nature of the laser-heated areas. The reader can learn the basics of this technique, which are presented in Midgley’s work [43].

**Figure 8:**
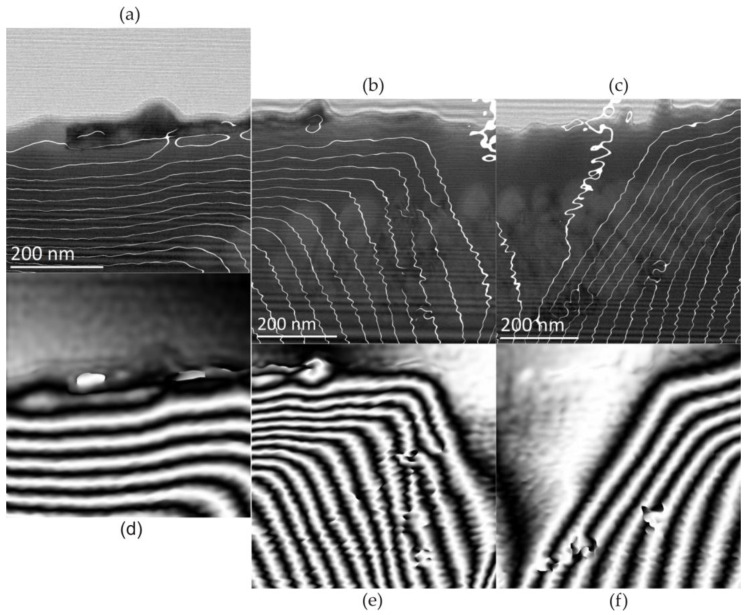
The images show the electron holograms acquired for the Fe_77_Cu_1_Si_13_B_9_ ribbon after laser heating in the absence of an external field. The magnetic field lines are shown (**a**) in the amorphous matrix; (**b**,**c**) phase shift of the magnetic field lines in the area of nanocrystallite clusters, in the absence of magnetic field excitation (H); (**d**–**f**) superimposed images of magnetic field lines into a microscopic image. The image refers to the isolated area in Figure 6. Nanocrystallites (α-Fe(Si)) disrupt the position of field force lines.

Figure 8a shows a hologram of the selected peripheral area of the dot in a plane perpendicular to the heating surface (Figure 6) with grouped nanocrystallites embedded in an amorphous matrix.

In Figure 8, changes in the magnetic field lines of force depending on the applied magnetic field are shown. In the absence of an external magnetic field, the recorded hologram, in the area of the amorphous matrix, shows characteristically parallel lines of the magnetic field to the edge of the sample (Figure 8a). In turn, the bending of the magnetic field lines occurred in the area of the crystalline phase (Figure 8b,c). Therefore, crystallites cause a step change in the magnetic induction vector. This is due to magnetocrystalline anisotropy, i.e., the presence of crystal axes of easy and hard directions of magnetisation. In this case, the axis of easy magnetisation of the crystalline phase was directed perpendicularly to the edge of the sample. In the observed area, local discontinuities in the field force lines are also visible, which may be caused by the presence of crystallites with different crystallographic orientation.

**Figure 9:**
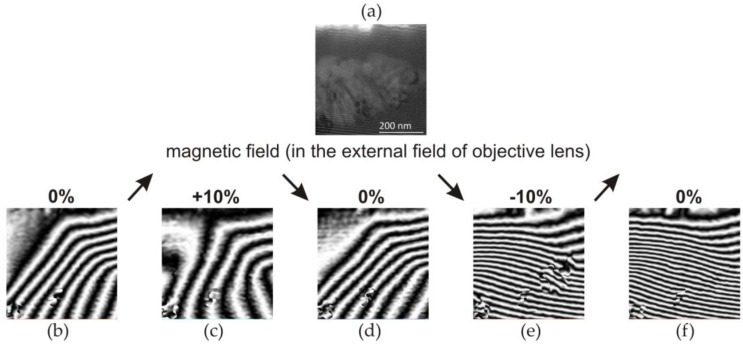
Images showing electron holograms of the Fe_77_Cu_1_Si_13_B_9_ ribbon after laser heating, recorded using an external variable magnetic field, obtained by objective lenses and by applying an excitation of ±10%. (+) and (−) correspond to the direction of current flow. (**a**) HRTEM image of nanocrystallite clusters in an amorphous matrix. Holograms correspond to (**b**) no external field; (**c**) +10%; (**d**) 0%; (**e**) −10%; and (**f**) 0%—no external field. The arrows symbolize changes in the current flowing through the objective lens.

Additionally, studies of the distribution of magnetic field forces in micro-areas of the laser-heated Fe_77_Cu_1_Si_13_B_9_ ribbons were carried out using an external magnetic field. Electron holograms were obtained by inducing a magnetic field with the objective lens of a microscope by flowing ±10% of the maximum lens current. Two types of excitations were performed using the direction of current flow: clockwise (+10%) and anticlockwise (−10%). The recorded electron holograms of the Fe_77_Cu_1_Si_13_B_9_ sample subjected to a magnetic field are shown in Figure 9. Figure 9b–f show holograms recorded according to the following excitation plan with objective lenses: initial state of 0% excitation (Figure 9b), with +10% excitation (Figure 9c), without excitation, 0% (Figure 9d), with excitation −10% (Figure 9e), and without excitation (Figure 9f).

Considering the magnetisation with a positive magnetic field (+10%), one should notice a slight change in the distribution of magnetic field forces (Figure 9c). However, the distance between the lines changed, and thus, the value of the magnetic field changed. After the external magnetic field (Figure 9d), the sample returned to its initial state (Figure 9b). In the next step, the application of an external magnetic field with the opposite direction (negative (−10%)) of the current flow resulted in the remagnetisation of both the amorphous matrix and the crystallite clusters (Figure 9e). There was a change in the direction of the magnetic field, and its intensity increased, observed through a changing arrangement of magnetic force lines in crystalline areas. The shutoff of the external magnetic field (Figure 9f) resulted in the return of the characteristic magnetisation of the foil to the level characteristic of the initial state. This means that the external magnetic field generated at −10% excitation led to strong overmagnetisation of the amorphous matrix as well as the nanocrystallites. It should be stated that the distributions of the magnetic field lines of force created in the absence of lens excitation (0%) show the magnetic remanence of the material (Figure 9b,d,f). Moreover, as can be seen, the remanence after negative excitation with lenses (−10%) is characterised by a much stronger magnetisation of the matrix. The analyses of the results lead to the conclusion that the easy direction of magnetisation of crystallites is probably such that the external magnetic field created at +10% excitation does not cause their magnetisation. Only the amorphous matrix is magnetised and returns to its initial state after the magnetic field is turned off. In the case of a field created by the negative excitation of the lens, both the matrix and the crystallites are magnetised. After the magnetic field, even when the matrix magnetisation disappears, the crystallites remain magnetised, and they are probably the ones that magnetise the matrix.

## 4. Discussion

The influence of pulsed laser interference heating (PLIH) on the change in the microstructure characteristics of the surface and near-surface areas of an amorphous Fe_77_Cu_1_Si_13_B_9_ ribbon is presented. Our research shows that the advantage of PLIH is the ability to heat specific areas of the ribbon surface in a very short time of laser beam operation. PLIH is a technique that can be applied to other amorphous materials. Thus, it is then necessary to select individual process parameters (e.g., energy, number of pulses, etc.) in order to achieve local heating of the material.

The laser heating of amorphous Fe_77_Cu_1_Si_13_B_9_ ribbons allowed for the simple and rapid creation of 62,500 dots with a diameter of approximately 10 µm, periodically arranged (at a distance of Λ = 17 µm) on their surface (Figure 3). Obtaining 62,500 dots in a specific area (5 × 5 mm) treated with a laser beam is an important aspect of the local heating of the material because reducing the distance between the dots would lead to their overlap. Such treatment causes the cumulative heating of micro-areas, which can result in the melting and ablation of the material (evaporation). Intensification of the process and the supply of large amounts of energy increase the level of thermal stresses that lead to the cracking of the material. At the same time, reducing the number of points in the mentioned area would lead to a smaller number of nanocrystallites embedded in the amorphous matrix and, consequently, to a deterioration in the assumed magnetic properties.

The laser beam is characterised by a Gaussian energy distribution; thus, the highest temperature is reached in its centre and decreases with the distance from its centre to the amorphous matrix. This results in structural changes obtained in each of the 62,500 dots. Depending on the pulsed laser interference heating parameters, one can ‘saturate’ the surface layer of an amorphous alloy with nanocrystals. Due to the mentioned Gaussian energy distribution in the laser beam, the structure in the dots will be different in the dot centre (usually nanocrystals) and dot peripheral areas (nanocrystals embedded by an amorphous matrix). This leads to the stabilisation of the structure and does not affect the crystallisation temperature of the alloys but may probably modify the Curie temperature.

SEM observations of the ribbon surface after laser heating showed that the use of the lowest energy and number of laser beam pulses led only to the heating of very thin surface layers of the ribbons (up to 100 nm in depth). An increase in the energy (above 120 mJ) and the number of pulses above 200 (Figure 4a) resulted in the accumulation of energy in the heated micro-areas, leading to the melting and ablation of the material. Explosive evaporation of the material occurs as a consequence of exceeding the ablation threshold. However, heating the laser beam with energy above 170 mJ (Figure 4c), regardless of the number of pulses, led to melting and laser ablation. The interaction of the laser beam with the ribbon material led to high temperatures reaching the solidus range only in its thin subsurface layer and crystallisation of the material to a depth of 350 nm.

In the process of preparing thin foil, the material was cut in a plane parallel to the laser-heated surface of the ribbon and ground on the heated side due to its greater surface roughness obtained as a result of the impact of the laser beam. Then, its central part was electrolytically polished and ion-cleaned. The position of the thin foil in relation to the zones formed during heating is shown in Figure 1 (red line). As a result, the thin foil cut through four characteristic zones in cross-section (Figure 5): (1) the centre of the dot, where nucleation and growth of the crystalline phase occurred, resulting in a granular structure; (2) the edge, where the crystallite growth rate was higher than the nucleation rate, leading to the formation of a coarse-grained structure; (3) a ring which was quickly melted and cooled and in which the material amorphised; and (4) the edge where the nucleation of a large number of crystallites was evenly distributed in the amorphous matrix. The occurrence of these zones is determined by the Gaussian distribution of energy in the laser beam, which leads to the formation of a temperature gradient from the central zone of the micro-area to its peripheral zone. In the middle zone, an increase in the number of pulses led to an increase in the amount of accumulated heat, which promoted the formation of a large number of crystal nuclei and led to the formation of a structure with a grain size of approximately 50–250 nm (Figure 5a) [4,44,45]. The formation of a large number of crystallites led to the mutual blocking of their growth and thus to a coarse-grained structure.

**Figure 10:**
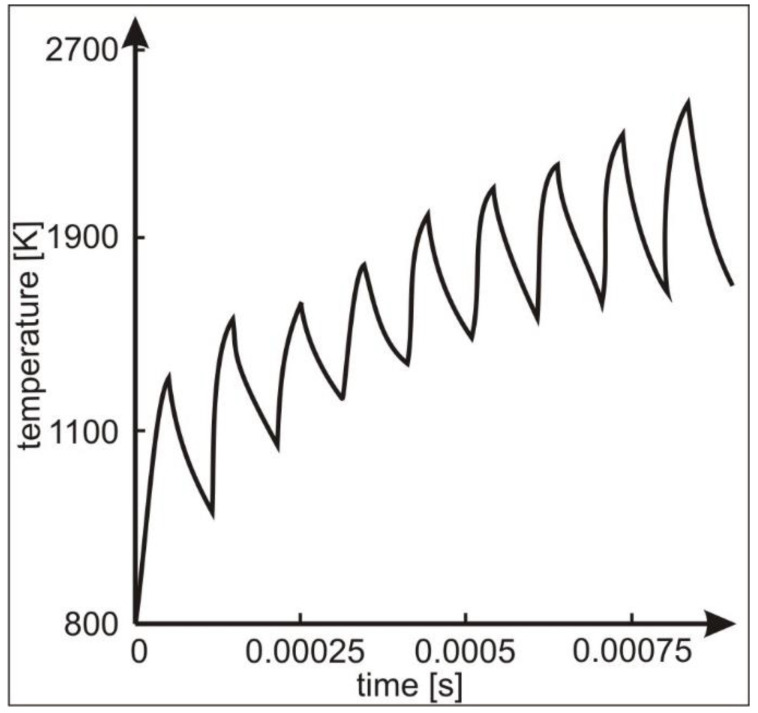
Scheme of surface temperature change in the pulsed-laser-heated micro-area. Based on [44].

As we moved away from the centre of the micro-area, the amount of energy decreased, resulting in fewer crystallisation nuclei being formed, and heating with subsequent pulses promoted their growth (grains up to approximately 1 µm were formed—zone 2, Figure 5a). A zone with an amorphous structure was formed around this zone (Figure 5a), and in the farthest peripheral zone, single or nanocrystallite clusters were formed, which were surrounded by an amorphous phase (Figure 5b). The probable mechanism for the formation of the amorphous structure is the accumulation of liquid material in this area, transferred by the shock wave (under the influence of centrifugal force) from the central part of the micro-area melted by the laser beam. The accumulation of liquid material, as a result of heat conduction, could lead to deeper melting of the ribbon in this area, and after the heating cycle stopped, it could lead to its vitrification again (Figure 10) [44]. The schema (Figure 10) corresponds to the amount of heat accumulated in the central part of the heated micro-area. Due to the pulsed nature of laser processing, as the number of pulses increases, there is an increase in the temperature in the processed area. Impulse interactions cause an ‘oscillatory’ cycle of rapid heating and cooling of the material. Each subsequent impact of the pulse leads to an increase in the temperature of the material, which is manifested by its sudden heating. Heat accumulation may also lead to the melting of the material.

Similar structural changes were also discovered in our previous structure obtained in FeSiB ribbons [6,34]. The presence of copper in the Fe_77_Cu_1_Si_13_B_9_ alloy led to conditions that favoured nucleation rather than the growth of existing grains, as confirmed by Kulik [23].

Due to the rapid heating of the material, the temperatures for the transformations in the solid state increase, and rapid solidification (a large number of crystallisation nuclei) allows for the formation of a crystalline structure composed of α-Fe(Si) crystals (Figure 4b and Figure 5). At the same time, copper plays a decisive role in the determination of nanocrystalline microstructures. The addition of copper to the FeSiB alloy causes chemical heterogeneity by forming clusters of Cu atoms, thus providing a high density of nucleation sites in the first stage of crystallisation of α-Fe(Si) nanocrystallites. During the impact of the laser beam, when the surface temperature of the ribbon increased to solidus temperature, the material was heated to high temperatures and crystallisation of the amorphous structure occurred, resulting in the formation of nanocrystallites (α-Fe(Si)) (Figure 6). At the same time, the short duration of the pulse and the cooling of the material led to a sudden ‘freezing’ of the structure. Furthermore, the action of subsequent shots caused the nucleation of new nanocrystalline grains without visible growth of the previously formed grains. It should be noted that at the same time, in the case of the FeSiB alloy without the addition of copper, the increase in energy and the number of pulses led to energy accumulation and, consequently, to grain growth [4,5,6]. However, the appearance of ripples in the peripheral area denotes the crystallisation of the amorphous material, creating nanocrystals grouped in the form of ‘wedges’ in this zone. Due to the presence of the nanocrystalline structure, the magnetisation of the ripples area is to be expected compared to the amorphous matrix, as confirmed by the magnetic force microscopy studies discussed in our previous work [6,33].

Our research highlighted the importance of electron holography (Figure 8), which was used to determine the nanoscale distribution of magnetic field lines in the micro-areas of laser-heated amorphous Fe_77_Cu_1_Si_13_B_9_ ribbons. This research was carried out using an HRTEM FEI Titan CUBED 80–300 microscope in Lorentz mode.

The structural changes that occur in the surface layers of Fe_77_Cu_1_Si_13_B_9_ ribbons as a result of the impact of PLIH influenced the distribution of the magnetic field lines of force (Figure 9). There was slight deflection of the magnetic field lines in the areas of small crystallites (α-Fe(Si)) embedded in the amorphous Fe_77_Cu_1_Si_13_B_9_ matrix. Magnetisation with a positive magnetic field (clockwise +10%) allowed only a slight change in the distribution of the magnetic field force lines and low magnetisation of the crystallites and the amorphous matrix. Therefore, it should be concluded that the easy direction of the magnetisation of the crystallites is different with positive excitation; therefore, the +10% excitation did not cause their magnetisation (Figure 9c). However, excitation with a negative magnetic field led to strong overmagnetisation of the nanocrystallites and the amorphous matrix (Figure 8e). The matrix was then magnetised as a result of the application of an external magnetic field (excitation by the objective lenses of the microscope), as well as by the crystallites present in it.

Local laser annealing of materials leads to controlled heating of the subsurface layers of the ribbons, which can significantly improve the plastic and magnetic properties compared to conventionally annealed ribbons. Conventional heating of the entire volume of the ribbon resulted in its almost complete crystallisation. This is undesirable because of the deterioration of the magnetic properties. However, as reported in our works [6,33], Fe_77_Cu_1_Si_13_B_9_ ribbons subjected to laser heating showed a higher saturation magnetisation (170 emu/g) compared to the material subjected to conventional crystallisation (156 emu/g) and compared to the material provided as an amorphous ribbon (163 emu/g).

To our knowledge, the stress level in the case of Fe_77_Cu_1_Si_13_B_9_ ribbons treated with PLIH has not been reported in the literature so far; this may be the direction of our future research. However, it can be assumed that the formation of compressive stresses is related to the appearance of a structural transformation. Therefore, during rapid cooling, the material shrinks, leading to reverse stress. However, because of the Gaussian energy distribution in the laser beam, the temperature distribution in the heated area changes from the highest (in the dot centre) to room temperature (in the peripheral). Thus, an unheated amorphous matrix relaxes most of the stresses.

## 5. Conclusions

The results of the conducted research broaden our understanding of determining the impact of pulsed laser interference heating on the formation of amorphous Fe_77_Cu_1_Si_13_B_9_ ribbons. The following conclusions can be drawn from the results obtained from the studies.

Favourable selective laser heating of ribbon in a very short time led to the creation of periodically arranged micro-areas composed of 62,500 dots.

Laser heating contributed to structural changes in the ribbon on the surface of 17 µm and in the near-surface areas reaching into the ribbon approximately 350 nm.

The initiation of crystallisation as a result of the impact of the laser beam on the amorphous Fe_77_Cu_1_Si_13_B_9_ ribbon and the rapid cooling allow the structure to be saturated with α-Fe(Si) nanocrystallites.

The addition of copper to the FeSiB alloy promoted the nucleation of new crystallites (approximately 10 nm in size) in the amorphous matrix.

Laser heating caused the crystallisation of the amorphous matrix and the formation of α-Fe(Si) nanocrystallites with a size of approximately 10 nm and an orientation of [001].

Changes in the distribution of the magnetic field line force occurred in the areas of α-Fe(Si) nanocrystallites.

## Data Availability

Data are contained within the article.
